# Beyond treatment eligibility: rethinking theranostic targets

**DOI:** 10.3389/fonc.2026.1937646

**Published:** 2026-08-19

**Authors:** Christopher Montemagno, Benjamin Serrano, Benoit Paulmier

**Affiliations:** 1 Medical Biology Department, Centre Scientifique de Monaco, Monaco, Monaco; 2 Medical Physics Department, Princess Grace Hospital, Monaco, Monaco; 3 Department of Nuclear Medicine, Princess Grace Hospital, Monaco, Monaco

**Keywords:** longitudinal imaging, molecular imaging, precision oncology, radioligand therapy, target biology, theranostic target, theranostics

## 1 Introduction

Theranostics occupies a unique position in oncology because the same molecular target simultaneously enables disease visualization and therapeutic delivery. Unlike most anticancer strategies, where biomarkers primarily determine treatment eligibility, theranostic targets directly connect molecular imaging with targeted radionuclide therapy, making them the biological foundation of the theranostic paradigm. This target-driven paradigm has transformed the management of several malignancies and established theranostics as one of the most successful examples of precision oncology (1, 2).

Consequently, theranostic targets have traditionally been regarded as gatekeepers to treatment. Demonstrating sufficient target expression before therapy remains the cornerstone of patient selection and underpins current international recommendations for theranostic radioligand therapies (3, 4). This paradigm has been remarkably successful but has largely focused attention on the presence of a target before treatment initiation.

Recent advances in tumor biology, however, suggest that this view may be incomplete. Molecular target expression is increasingly recognized as a dynamic biological phenotype shaped by intrapatient heterogeneity, clonal evolution, therapeutic pressure and treatment-induced modulation rather than a fixed characteristic of the disease (5). In prostate cancer, longitudinal imaging studies have demonstrated that PSMA expression can change rapidly following androgen receptor pathway inhibition, with substantial variability observed between metastatic lesions within the same patient (6). Likewise, the PSMA flare phenomenon illustrates that temporal variations in tracer uptake may reflect biological adaptation rather than simply changes in tumor burden (7).

These observations invite a fundamental reconsideration of the role assigned to theranostic targets. Should molecular targets continue to be viewed primarily as biomarkers defining treatment eligibility, or should they increasingly be regarded as dynamic biological systems whose temporal evolution provides biologically meaningful information? We propose that theranostic targets should be viewed not only as biomarkers guiding treatment decisions, but also as dynamic biological entities whose longitudinal evolution may provide biologically meaningful information throughout the course of disease and therapy. Importantly, this perspective should not be confused with conventional treatment monitoring or response assessment. Whereas response assessment primarily seeks to determine whether therapy is effective by evaluating changes in tumor burden or disease progression, the present concept considers the therapeutic target itself as the primary object of biological investigation. In this framework, serial molecular imaging is viewed not only as a tool for evaluating treatment efficacy, but also as a means of characterizing longitudinal changes in therapeutic target biology under therapeutic pressure.

In this Opinion, we discuss how recent advances in target biology support this conceptual shift and explore its potential implications for the future development of theranostics.

## 2 The emerging biology of theranostic targets

The remarkable success of theranostics has been built upon the identification of molecular targets capable of selecting patients for radionuclide therapy. Consequently, clinical practice has naturally focused on confirming sufficient target expression before treatment initiation, establishing molecular imaging as the gateway to therapy (3, 4). This approach has transformed patient care and remains the cornerstone of contemporary theranostic practice. However, it has also largely confined the biological role of theranostic targets to a single time point: treatment eligibility.

Emerging evidence, however, suggests that this view is incomplete. Rather than representing immutable features of cancer cells, molecular targets are increasingly recognized as dynamic biological phenotypes shaped by tumor differentiation, clonal evolution, interactions with the tumor microenvironment and therapeutic pressure (5). Consequently, target expression should not be interpreted solely as a binary indicator of eligibility, but also as a biological readout reflecting the evolving state of the disease.

PSMA biology provides one of the clearest examples of this concept. Longitudinal studies have demonstrated that androgen receptor pathway inhibition can rapidly modify PSMA expression, with substantial heterogeneity observed both between patients and between metastatic lesions within the same individual (6). Likewise, the recently described PSMA flare phenomenon illustrates that therapeutic interventions may transiently increase PSMA PET tracer uptake, likely reflecting therapy-induced modulation of target expression rather than simply changes in tumor burden (7). More recently, prospective clinical data have shown that patients with an initially negative PSMA PET/CT may subsequently demonstrate sufficient target expression to become eligible for PSMA-targeted radioligand therapy, further emphasizing that target expression is not a fixed biological characteristic but can evolve over time (8).

While PSMA currently provides the clearest clinical illustration of this concept, emerging evidence suggests that similar principles may not be restricted to prostate cancer. Similar observations have been reported in patients with neuroendocrine tumors undergoing peptide receptor radionuclide therapy, where serial somatostatin receptor imaging has revealed spatial and temporal heterogeneity in target behavior during treatment (9, 10). More broadly, the continued expansion of theranostic targets—including fibroblast activation protein (FAP) and CXCR4—suggests that this conceptual framework may ultimately extend across multiple theranostic systems as these platforms continue to mature (11, 12).

Collectively, these observations suggest that theranostic targets represent more than static eligibility biomarkers. Instead, they should increasingly be viewed as evolving biological systems whose longitudinal characterization provides insight into the dynamic interaction between tumor evolution and therapeutic intervention.

## 3 Observing target biology over time

If theranostic targets are dynamic biological systems, the next logical question is whether these biological changes can be observed throughout treatment. Unlike most systemic anticancer therapies, theranostics offers a unique opportunity to repeatedly characterize the same molecular target during the course of therapy through serial molecular imaging, post-treatment SPECT/CT, lesion-level dosimetry and quantitative response assessment (3, 13). Consequently, theranostics provides access not only to a pretreatment biological snapshot, but also to a longitudinal view of target biology.

Importantly, this longitudinal characterization extends beyond conventional response evaluation. Increasing evidence indicates that metastatic lesions within the same patient may follow distinct biological trajectories during radionuclide therapy, revealing spatial and temporal heterogeneity that cannot be adequately captured by patient-level response assessment alone (9, 10). Similarly, the RECIP 1.0 framework recognizes that longitudinal changes observed on PSMA PET provide information beyond conventional anatomical response criteria by integrating changes in whole-body PSMA tumor burden together with the appearance of new lesions (14).

Post-treatment dosimetry further reinforces this concept. Differences in absorbed dose between lesions and across successive treatment cycles demonstrate that biological responses are neither spatially nor temporally uniform, while lesion-level dose-response analyses suggest that the biological behavior of a target cannot always be inferred from a single baseline assessment (15, 16).

Collectively, these developments provide, for the first time, the opportunity to observe the temporal biology of theranostic targets throughout treatment, extending the role of molecular imaging beyond conventional response assessment.

## 4 Redefining the role of theranostic targets

These observations do not challenge the current clinical paradigm, as baseline molecular characterization remains fundamental for patient selection and continues to underpin the remarkable success of radionuclide therapies (3, 4). Recent biological and imaging evidence nevertheless suggests that the role assigned to theranostic targets may be broader than initially envisioned.

Historically, molecular targets have primarily answered a single clinical question: who is eligible for treatment?

Emerging evidence suggests that they may progressively help answer another, equally important one: how does tumor biology evolve during treatment? In this perspective, longitudinal molecular imaging, post-treatment dosimetry and serial target characterization are no longer viewed solely as tools for monitoring therapeutic efficacy. They also become complementary approaches for exploring the biological evolution of the therapeutic target itself (9, 13, 15).

Importantly, this perspective should not be interpreted as advocating immediate modifications of current clinical practice. There is currently no evidence that adapting therapy according to temporal changes in target expression improves clinical outcomes. Whether temporal changes in target expression should influence therapeutic decisions remains unknown and will require prospective validation. Nevertheless, acknowledging that molecular targets evolve over time fundamentally changes the questions future research should address. Rather than asking only whether a target is expressed before treatment, future investigations should seek to understand how target expression changes over time, and what these temporal dynamics reveal about tumor biology during therapy (17, 18). Prospective longitudinal studies integrating serial molecular imaging, dosimetry, and clinical outcomes will be essential to determine whether temporal changes in target biology carry prognostic or therapeutic significance.

Ultimately, the future of theranostics may depend not only on discovering new molecular targets, but also on fully understanding the biological behavior of those already available. In this evolving framework, theranostic targets become more than gateways to therapy; they become longitudinal biological readouts capable of providing new insight into the interaction between tumor evolution and therapeutic intervention.

## 5 Conclusion

The development of theranostics has largely been driven by the discovery of molecular targets linking diagnosis and therapy. Accumulating evidence now suggests that these targets represent more than biomarkers for treatment eligibility, instead behaving as evolving biological phenotypes whose expression reflects the interaction between tumor biology and therapeutic pressure.

This conceptual shift does not call current clinical practice into question. Instead, it broadens the framework through which theranostic targets are interpreted and opens new avenues for biological investigation. As longitudinal imaging, dosimetry and computational approaches continue to mature, understanding not only where a target is expressed, but also how and why it evolves over time, may ultimately redefine the role of theranostic targets in precision oncology.
